# Investigating the association between tobacco use and oral health among security guards at a tertiary healthcare centre in New Delhi: a cross-sectional study

**DOI:** 10.3389/froh.2024.1375792

**Published:** 2024-07-10

**Authors:** Neha Chauhan, Sarah Paul, Upendra Singh Bhadauria, Bharathi M. Purohit, Ritu Duggal, Manali Deb Barma, Deepali Agarwal, Anuradha Bhukal, S Sasidharan, Pallavi Shukla, Maroof Khan, Ramya Shenoy, Sumit Malhotra, Harsh Priya

**Affiliations:** ^1^ Centre for Dental Education and Research, All India Institute of Medical Sciences, New Delhi, India; ^2^ Division of Public Health Dentistry, Centre for Dental Education and Research, All India Institute of Medical Sciences, New Delhi, India; ^3^ Division of Orthodontics and Dentofacial Deformities, Centre for Dental Education and Research, All India Institute of Medical Sciences, New Delhi, India; ^4^ Department of Preventive Oncology, Dr. B.R.A Institute-Rotary Cancer Hospital, All India Institute of Medical Sciences, New Delhi, India; ^5^ Department of Bio-Statistics, All India Institute of Medical Sciences, New Delhi, India; ^6^ Department of Public Health Dentistry, Manipal College of Dental Sciences, Mangalore, India; ^7^ Centre for Community Medicine, All India Institute of Medical Sciences, New Delhi, India

**Keywords:** security guards, tobacco usage, smokeless tobacco (SLT), smoking tobacco, oral potentially malignant disorders (OPMDs), gender disparities, occupational health, public health

## Abstract

**Background:**

Tobacco usage is a major global public health concern, contributing to millions of deaths annually. This study focuses on security guards, an occupational group facing unique challenges, to investigate tobacco prevalence, usage patterns, and associated oral health risks.

**Methods:**

A cross-sectional study was conducted among security guards at a Tertiary Health Care Centre, from October 2022 to February 2023. Data on demographics, tobacco habits, and comorbidities were collected via a structured questionnaire. Clinical oral examinations provisionally diagnosed Oral Potentially Malignant Disorders (OPMDs) based on clinical findings. Appropriate Statistical analyses were employed.

**Results:**

Among 696 security guards, 40.1% used tobacco, including 15.0% smokers and 74.5% engaging in smokeless tobacco. Additionally, 10.3% reported using both smoking and smokeless forms. Non-tobacco users accounted for 59.9%. Tobacco users showed a higher prevalence of OPMDs (11.4%) compared to non-tobacco users (1.4%).

**Discussion:**

Security guards demonstrated a higher tobacco prevalence, with smokeless tobacco being predominant. Gender disparities in tobacco use emphasize the need for gender-specific interventions. The study highlights the significant impact of tobacco on oral health, especially the risk of OPMDs.

**Conclusion:**

A high prevalence of tobacco usage (40.1%), particularly smokeless tobacco, among security guards, emphasizes the importance of targeted interventions within this occupational group. Oral Potentially Malignant Disorders (OPMDs) were significantly more prevalent (11.4%) in tobacco users. The association between tobacco usage and OPMDs reaffirms the well-established association between tobacco and adverse oral health outcomes.

## Introduction

The severity of the global public health challenge posed by tobacco is one of the most significant issues humanity has encountered, resulting in the loss of around 8 million lives every year. Over 7 million of these fatalities trace back to direct tobacco utilization, while approximately 1.3 million result from non-smokers being exposed to the effects of second-hand smoke (1). In recent times, it has become evident that the threat of encountering tobacco smoke doesn't dissipate once tobacco products are no longer in use. Instead, it lingers even in the absence of these products and can persist on various surfaces for extended periods, sometimes lasting for weeks or even up to 1.5 years, as reported in the case of THS (Thirdhand Smoke). This newly recognized concept of Third-hand Smoke is particularly concerning for infants and young children, who are more vulnerable due to their rapid breathing, thinner skin, and increased time spent in areas where dust containing THS has accumulated (2).

Every type of tobacco usage carries inherent risks, and there exists no level of tobacco exposure that can be deemed safe. Among these, cigarette smoking stands as the prevailing form of tobacco consumption globally. Additional tobacco items encompass waterpipe tobacco, cigars, cigarillos, heated tobacco, self-prepared roll tobacco, pipe tobacco, bidis, kreteks, and smokeless tobacco products (3).

Tobacco is among the substances that are most readily obtainable, legally sanctioned, and conveniently accessible. Approximately 80% of the 1.3 billion global tobacco consumers reside in countries with lower to middle income. During the year 2020, tobacco usage was prevalent among about 22.3% of the global population, encompassing 36.7% of men and 7.8% of women (3). In India according to The Global Adult Tobacco Survey 2 (GATS 2) 2016–2017, among adults, 19.0% of men, 2.0% of women, and a total of 10.7% (99.5 million) currently engage in smoking tobacco. Furthermore, 29.6% of men, 12.8% of women, and a combined 21.4% (199.4 million) currently use smokeless tobacco. Overall, 42.4% of men, 14.2% of women, and a collective 28.6% (266.8 million) of all adults currently engaged in tobacco consumption, encompassing both smoking and smokeless forms (4).

Smoking is a contributing factor to early mortality linked with prevalent chronic conditions, including cardiovascular ailments, cancer, diabetes, and chronic respiratory disorders. Smoking is accountable for the loss of 2,060,000 years of healthy life, representing 16.3% of the total disability-adjusted life year (DALY) measurement (5). Chewing tobacco and similar smokeless tobacco products are recognized to have adverse effects on oral health. The major types of oral mucosal soft-tissue lesions induced by smokeless or chewing tobacco include oral squamous cell carcinoma and verrucous carcinoma, potential precancerous disorders (leukoplakia, erythroplakia, and erythroleukoplakia), and tobacco pouch-related lesions (seen in tobacco and lime users, or combined with areca nut leading to oral submucous fibrosis) (6). The International Agency on Research for Cancer (IARC) has confirmed that smokeless tobacco is a known human carcinogen, with a specific impact on the orally applied region (7). A meta-analysis of 10 studies demonstrated a 6.19 adjusted relative risk (confidence interval: 4.16–9.21) for the development of oral and oropharyngeal cancer associated with the consumption of betel quid combined with tobacco for chewing (8).

Tobacco utilization isn't just about personal choices; constituting a multifaceted interplay of sociocultural, environmental, and psychological influences. Empirical data underscore the propensity for tobacco initiation to occur during adolescence (9). Intrinsically, determinants including gender, socioeconomic status, familial propensities, peer behaviors, advertising exposure, and residential context contribute to this complex behavior (10). Research studies have identified smokeless tobacco use was found to be significantly linked with factors such as occupation, employer type, workplace location, business nature, and workload (11, 12).

Security guards play a pivotal role in ensuring safety across diverse environments, including hospitals, where their responsibilities often involve extended and nighttime shifts, as well as managing potentially stressful situations. These factors may contribute to unhealthy habits, such as tobacco use. Studies have documented variations in the prevalence of tobacco use among security guards. For instance, Kalyani et al. reported an overall prevalence of 45.7% of tobacco use among security guards, with a notable emphasis on smokeless tobacco use (13). Conversely, Gaunkar R et al. found a lower prevalence of 15.9%, indicating healthier tobacco consumption practices among security guards in their study. They attributed this to good practices and awareness of tobacco control laws, suggesting that despite potential stress and irregular working hours, security guards demonstrated better tobacco consumption habits (14).

Despite increasing awareness of tobacco’s adverse health effects, there remains a gap in our understanding of its prevalence and patterns among security guards. This study is essential for several reasons. Investigating tobacco usage habits within this occupational group helps assess the impact on security guards’ health, provides insights into factors contributing to tobacco usage, and informs targeted interventions and policies. Understanding tobacco usage patterns among security guards is foundational for developing customized workplace-based cessation programs tailored to their unique needs. Healthcare institutions are fundamental components of a society's health infrastructure, serving as primary providers of preventive and treatment services. A tobacco-free hospital environment is a vital step in promoting overall health, decreasing susceptibility to tobacco use, boosting cessation rates, and influencing patient's attitudes toward tobacco consumption. Security guards can play a pivotal role in maintaining this tobacco-free campus by enforcing policies, monitoring compliance, and educating visitors about the benefits of a tobacco-free environment. Through their vigilance and proactive engagement, security guards can contribute significantly to fostering a healthier and tobacco-free atmosphere within healthcare facilities.

The objectives of this study were: (i) To assess the prevalence of tobacco usage among security guards, including smoking and smokeless tobacco use. (ii)To establish the association of demographic factors, and clinical findings with tobacco usage among security guards(iii) To find out the prevalence of OPMDs among security guards (iv) To find the association between demographic factors, clinical findings, and the oral potentially malignant disorders (OPMDs) among security guards.

## Methodology

### Study setting and duration

A cross-sectional study was conducted at the Centre for Dental Education and Research (CDER) within the All India Institute of Medical Sciences (AIIMS) campus in New Delhi. The study took place at the Tobacco Cessation Clinic situated on the ground floor of CDER, AIIMS, New Delhi, spanning from October 2022 to February 2023.

### Ethical considerations

Before the start of the study, the purpose and procedure of the study were explained and permission was obtained from the concerned authority of AIIMS, New Delhi. The study was approved by the institutional review board committee, and all participants provided written informed consent. The study was also registered with the Clinical Trials Registry of India (CTRI) with reference number REF/2020/09/058387.

### Sample size determination

During a pilot study involving 30 security guards, the prevalence of tobacco usage was found to be 24%. Based on this pilot test prevalence, with a 95% confidence level and a margin of error of 3%, the final sample size was determined to be 683. A total of 696 security guards participated in the study.

### Inclusion and exclusion criteria

Inclusion criteria for participant selection comprised security guards specifically appointed at AIIMS, New Delhi. Exclusion criteria included security guards who did not provide consent to participate in the study.

### Sampling method

The sampling method employed for participant selection was simple random sampling, ensuring equal opportunity for all eligible security guards to be included in the study.

### Operational definitions of OPMDs

During the clinical examination, the examiner made provisional diagnoses of OPMDs based on clinical findings. The operational definition of OPMDs encompasses a range of oral mucosal abnormalities associated with an increased risk of oral cancer development. These disorders included leukoplakia, oral submucous fibrosis (OSMF), erythroplakia, tobacco pouch keratosis, lichen planus, smoker's palate, and other abnormalities suggestive of premalignant changes or the absence of identifiable disorders.

### Clinical examination

Two trained examiners conducted clinical oral examinations for Oral Potentially Malignant Disorders (OPMDs), ensuring enhanced reliability and validity. Each examiner independently assessed participants’ clinical findings, facilitating the assessment of inter-rater reliability, which was found to be substantial (Kappa coefficient = 0.85).

### Data collection

Data on socio-demographics, tobacco usage patterns, and associated risk factors were collected using a structured and validated questionnaire through the interview method. The questionnaire underwent thorough validation, including pilot testing and expert review, to ensure clarity and relevance. It achieved satisfactory validity and reliability, with a Cronbach's alpha coefficient exceeding 0.70.

Non-Tobacco Users: Individuals who answered “No” to questions about smoking or using smokeless tobacco products. Smokers: Individuals who answered “Yes” to current smoking, irrespective of their intention to quit. Smokeless Tobacco Users: Individuals who answered “Yes” to current use of smokeless tobacco products.

Study subjects with tobacco usage were informed and educated regarding cessation through the Tobacco Cessation Clinic at CDER, AIIMS, New Delhi. This initiative aimed to provide comprehensive support and guidance to individuals seeking to quit tobacco use, encompassing counseling, behavioral interventions, and pharmacotherapy where appropriate.

### Data analysis

Using the Statistical Package for the Social Sciences (SPSS) software program, version 21.0, the data were coded, tabulated, and subjected to the necessary statistical analysis. Analytic statistics and descriptive statistics (frequency distribution) were applied to the data analysis. To assess the relationship between independent variables and tobacco usage, the chi-squared test was conducted. The significance level was set at *p* < 0.05% and 95% confidence interval.

A multivariable logistic regression model was constructed with the OPMDs condition as the dependent variable. Variables included in the model were age, gender, comorbidities, and medication users.

### Reporting standards

In accordance with best practices for observational studies, this study adhered to the STROBE (Strengthening the Reporting of Observational Studies in Epidemiology) guidelines to ensure comprehensive and transparent reporting of methods and results.

## Results

Among 696 security guards 279 (40.1%) security guards were identified as tobacco users, while 417 (59.9%) were non-tobacco users. 42 (15.0%) security guards were identified as smoking tobacco users. The majority, constituting 208 (74.5%) were engaged in smokeless tobacco use, 29 (10.3%) of the security guards reported using both smoking and smokeless forms of tobacco (Table 1).

**Table 1:** Prevalence of tobacco usage among security guards.

Total number of security guards (*N* = 696)	Frequency (%)
Tobacco users	279 (40.1%)
Non-tobacco users	417 (59.9%)
Tobacco users (*N* = 279) Tobacco forms	
Smoking tobacco users	42 (15.0%)
Smokeless tobacco users	208 (74.5%)
Both forms of tobacco users	29 (10.3%)

The mean age of tobacco users was 38.52 ± 9.11, and for non-tobacco users, it was 37.32 ± 8.44 years (*p*-value = 0.389). Mean Tobacco usage Initiation age was 24.5 ± 11.06 years. Among tobacco users, 275 (39.5%) were male and 4 (0.6%) were female, while for non-tobacco users, 286 (41.0%) were male, and 131 (18.8%) were female (*p* < 0.001). The presence of various comorbidities was examined in both groups: 12 (1.7%) tobacco users and 15 (2.2%) non-tobacco users had diabetes, 19 (2.7%) tobacco users and 13 (1.8%) non-tobacco users had hypertension, and 1 (0.14%) tobacco users and 1 (0.14%) non-tobacco users had cardiac disease. Additionally, 2 (0.3%) tobacco users and 1 (0.14%) non-tobacco users had respiratory disease. There was no statistically significant difference in comorbidities between the two groups (*p* = 0.07). The assessment of medication use showed no significant association with tobacco usage (*p* = 0.501). The mean duration of past quitting attempts among individuals who are currently using tobacco was 1.6 ± 2.6 years. Among tobacco users, 80 (11.4%) had OPMDs, while only 10 (1.4%) non-tobacco users presented with these disorders (*p* < 0.001). Leukoplakia was identified in 16 (0.8%) tobacco users and 3 (0.7%) non-tobacco users. Similarly, OSMF (Oral Submucous Fibrosis) was found in 7 (0.14%) tobacco users and 1 (0.2%) non-tobacco user. Tobacco Pouch Keratosis was found in 54 (7.7%) tobacco users and 1 (0.2%) non-tobacco user. The occurrence of Smoker's Palate was found in 3 (0.43%) tobacco users and 0 (0.0%) non-tobacco users. OPMDs, including Leukoplakia, OSMF, Tobacco Pouch Keratosis, and others, revealed a significantly higher prevalence among tobacco users compared to non-tobacco users (*p* < 0.001) (Table 2).

**Table 2:** Association of demographic factors, and clinical findings with tobacco users and non-tobacco users among security guards.

Variables	Tobacco user	Non-tobacco users	Chi-square/fisher exact	*p*-value
Age (years) Mean ± S.D	38.52 ± 9.11	37.32 ± 8.44	** **	0.389
Gender				
Male	275 (39.5%)	286 (41.0%)	*χ*^2^ = 96.106, df = 1	**<0**.001**
Female	4 (0.6%)	131 (18.8%)		
Comorbidities			*χ*^2^ = 8.906, df = 5	0.07
Diabetes	12 (1.7%)	15 (2.2%)		
Hypertension	19 (2.7%)	13 (1.8%)		
Cardiac disease	1 (0.14%)	1 (0.14%)		
Respiratory disease	2 (0.3%)	1 (0.14%)		
Any other	7 (1.0%)	21 (3.01%)		
None	238 (34.1%)	366 (52.5%)		
Comorbidities			*χ*^2^ = 0.886, df = 1	0.34
Present	41 (5.9%)	51 (7.3%)		
Absent	238 (34.1%)	366 (52.5%)		
Medication users			*χ*^2^ = 0.505, df = 1	0.50
Yes	35 (5.02%)	45 (6.46%)		
No	244 (35.1%)	372 (53.4%)		
Duration of medication (Years) Mean ± S.D	4.68 ± 5.78	3.51 ± 3.47		0.06
Age of initiation of tobacco usage (Years) Mean ± S.D	24.5 ± 11.06			
Duration of past quitting attempts (Years) (Currently using) Mean ± S.D	1.6 ± 2.6			
Oral potential premalignant disorders			*χ*^2^ = 119.67, df = 5	**<0**.001**
Leukoplakia	16 (0.8%)	3 (0.7%)		
OSMF	7 (0.14%)	1 (0.2%)		
Erythroplakia				
Tobacco pouch keratosis	54 (7.7%)	1 (0.2%)		
Lichen planus				
Smoker's palate	3 (0.43%)	0 (0.0%)		
Any other	1 (0.14%)	5 (1.2%)		
None	199 (28.5%)	407 (58.4%)		
Oral potential premalignant disorders			*χ*^2^ = 102.5, df = 1	**<0**.001**
Present	80 (11.4%)	10 (1.4%)		
Absent	199 (28.5%)	407 (58.4%)		

Independent *t*-test.

* *p *< 0.05 is significant, Chi-square/fisher exact Test.

** *p *< 0.05 is significant.

Age was found to have a significant association with the prevalence of OPMDs, with an odds ratio (OR = 1.0, *p *= 0.01, CI =1.00–1.05), indicating that for each year increase in age, the odds of having OPMDs increased by a factor of 1.0. Gender exhibited a significant association, with male security guards having significantly higher odds (OR = 8.1, *p* < 0.01, CI = 2.51–25.9) of having OPMDs compared to their female counterparts. Among comorbidities, hypertension was notably associated with OPMDs, (OR = 2.4, *p* = 0.03, CI = 1.05–5.59) signifying that security guards with hypertension had 2.4 times higher odds of having OPMDs than those without this condition. The analysis also considered the role of tobacco usage. Smoking, smokeless tobacco use, and using both forms of tobacco were all significantly associated with a higher likelihood of having OPMDs, with ORs of 5.5 (*p* = 0.003, CI = 1.78–16.9), 18.0 (*p* < 0.001, CI = 9.04–36.1), and 24.8 (*p *< 0.001, CI = 9.35–66.1), respectively. The confidence intervals for the different forms of tobacco use, including smoking, smokeless tobacco, and both forms, vary in width due to differences in sample sizes and the prevalence of these tobacco habits among the study population. Additionally, the duration of past tobacco quitting attempts showed significance, with an OR of 0.7 (*p* = 0.05, CI = 0.48–1.02), suggesting that a longer duration of tobacco usage quitting was associated with lower odds of having OPMDs. (Table 3). Multivariable analysis was conducted with the significant factors identified in the univariable analysis, and similar associations persisted even after adjusting for age and tobacco usage, indicating significant relationships between these factors and the presence of OPMDs.

**Table 3:** Univariable analysis for the association between demographic factors, clinical findings, and the prevalence of oral potentially malignant disorders among security guards.

Variables	OPMDs prevalence	Odds ratio 95% confidence interval	*p*-value
Age(years)		1.0 (1.00–1.05)	**0**.**01**
Gender			
Male	87 (96.7%)	8.1** (**2.51–25.9)**	**<0**.**001**
Female*	3 (3.3%)	1.0	
Comorbidities			
Diabetes	6 (6.7%)	2.1 (0.81–5.31)	0.12
Hypertension	8 (8.9%)	2.4** (**1.05–5.59)**	**0**.**03**
Cardiac disease	0 (0.0%)		
Respiratory disease	1 (1.1%)	3.6 (0.32–40.6)	0.29
Any other	2 (2.2%)	0.5 (0.13–2.40)	0.43
None*	73 (81.1%)	1.0	
Comorbidities			
Present	17 (18.9%)	1.6 (0.92–2.94)	0.09
Absent*	73 (81.1%)	1.0	
Medication users			
Yes	15 (16.7%)	1.6 (0.90–3.06)	0.10
No*	75 (83.3%)	1.0	
Tobacco users			
Smoking	5 (5.6%)	5.5** (**1.78–16.9)**	**0**.**03**
Smokeless	64 (71.1%)	18.0** (**9.04–36.1)**	**<0**.**001**
Both	11 (12.2%)	24.8** (**9.35–66.1)**	**<0**.**001**
No*	10 (11.1%)	1.0	
Duration of comorbidities (years)		1.1 (0.97–1.18)	0.14
Age of initiation of tobacco use		0.9 (0.96–1.01)	0.58
Duration of past quitting attempts (Years) (Currently using)	** **	0.7** (**0.48–1.02)**	**0**.**05**

* Reference, **p < 0.05 is significant, OPMDs, oral potentially malignant disorders.

## Discussion

The study was conducted on a cohort of security guards, and the decision to select them as the study cohort is supported by their unique work setting. Security guards frequently encounter demanding job conditions marked by extended shifts, repetitive tasks, and the obligation to uphold safety and order. These stress-inducing factors can have a notable impact on how individuals respond, potentially prompting various coping strategies, including the use of tobacco. Investigating the prevalence of tobacco use within this specific cohort is essential, not only for gaining insights into how occupational settings influence their lifestyle decisions but also for devising targeted interventions aimed at enhancing their overall quality of life.

The overall prevalence of tobacco use among security guards was found to be 40.1%, with a significant gender disparity where male security guards exhibited a prevalence of 39.5%, while female security guards had a much lower prevalence of 0.6%. This suggests a significant gender difference in tobacco consumption patterns within this occupational group. Comparing these results to the Global Adult Tobacco Survey 2 (GATS 2) conducted in 2016–17, it is evident that the prevalence of tobacco use among security guards in the present study is notably higher than the national average. According to GATS 2, 28.6% of all adults in India currently consume tobacco in either smoked or smokeless form, with 42.4% of men and 14.2% of women engaging in tobacco use (4). The present study's findings indicate that security guards have a higher tobacco use prevalence, particularly among male security guards, which suggests that this occupational group might be more vulnerable to tobacco use compared to the general population.

In comparison to GATS 2 figures, the classification of tobacco use forms in the current study shows a significant variance. The prevalence of smoking tobacco users among security guards was 15.0%, which is considerably higher than the national prevalence of 10.38% for smoking tobacco use in India (4). Conversely, the prevalence of smokeless tobacco users among security guards was 74.5%, surpassing the national prevalence of smokeless tobacco use in India, which stands at 21.38% (4). This highlights a distinctive pattern of tobacco consumption among security guards, with smokeless tobacco being more predominant than smoking tobacco. In contrast to the present study's results, **Kalyani et al.** (13) reported a slightly higher overall prevalence of tobacco use among security guards (45.7%). Notably, **Kalyani et al.** (13) identified a higher prevalence of smokeless tobacco users (34%) among security guards, which is consistent with the present study's emphasis on smokeless tobacco use being the dominant form of tobacco consumption in this cohort. On the other hand, the study conducted by **Gaunkar R et al.** (14) reported a substantially lower prevalence of tobacco use among security guards (15.9%). This contradicts the present study's findings. **Gaunkar R et al.** (14) noted a lower prevalence of both smoking and smokeless tobacco users, indicating that the security guards in their study exhibited healthier tobacco consumption practices. This could be attributed to good practices and awareness among security guards despite the potential stress and irregular working hours associated with their jobs as 68.6% of the study participants were aware of any tobacco control law in India (14). In the present study, we observed a significant association between tobacco use and the occurrence of OPMDs among security guards. Specifically, 11.4% of tobacco users presented with OPMDs, while only 1.4% of non-tobacco users exhibited these disorders. The odds of having OPMDs were significantly higher among individuals who used tobacco in various forms (*p* < 0.001). This underscores the heightened risk of OPMDs associated with tobacco consumption within this occupational group. Comparing the findings with those of **Singh AK et al**. (15), who reported an OPMD prevalence ranging from 13.3% to 13.9% among the general population, and **Sekizhar V et al.** (16), who noted an OPMD prevalence of 16.79% among fishermen, the present study observed a similar trend of elevated prevalence among specific occupational cohorts. Notably, while the prevalence rates may vary across different populations, the study underscores the importance of recognizing the heightened risk of OPMDs among security guards, particularly attributable to tobacco use. The results of the present study provide empirical evidence supporting the biological mechanisms underlying the association between tobacco use and the development of various oral potentially malignant lesions. Among tobacco users, 80 (11.4%) exhibited Oral Potentially Malignant Disorders (OPMDs), a significantly higher prevalence compared to the 1.4% observed in non-tobacco users. The underscores the heightened risk of OPMDs associated with tobacco consumption. Specifically, tobacco usage as compared to non-tobacco usage was associated with a higher prevalence of pathologic lesions such as leukoplakia, oral submucous fibrosis (OSMF), tobacco pouch keratosis, and smoker's palate. These findings align with the known mechanisms by which smokeless tobacco exerts its detrimental effects on oral health. Smokeless tobacco contains chemical carcinogens such as benzo[a]pyrene, nicotine, and tobacco-specific N-nitrosamines, which can induce dysplastic changes in the oral mucosa. Chronic intraoral placement of smokeless tobacco leads to chronic irritation, triggering the deposition of excess fibrin-like material and an increase in keratin production, characteristic of lesions like smokeless tobacco keratosis. Leukoplakia and erythroplakia, both clinically diagnostic terms for oral lesions, can also arise from the carcinogenic effects of tobacco and serve as visible indicators of tissue damage and potential malignant transformation (17). In the case of OSMF, while areca nut-chewing is identified as the primary causative agent, the chewing of smokeless tobacco significantly contributes to its development. The chronic exposure to carcinogens and irritants presents in smokeless tobacco exacerbates fibrotic changes in the oral submucosa, leading to the characteristic stiffness and limited mouth opening associated with OSMF (18). The present study identified age as a statistically significant risk factor for OPMDs (OR = 1.0, *p* = 0.01). For each year increase in age, the odds of having OPMDs increased by a factor of 1.0. This finding is consistent with prior research, such as the study conducted by **Alessandro V et al.** (19), which also reported a significant association between increasing age and the prevalence of OPMDs. Another noteworthy observation was the significant gender disparity in OPMDs prevalence. Males exhibited substantially higher odds of having OPMDs compared to females (OR = 8.1, *p* < 0.01). This gender-based difference in OPMD prevalence is consistent with findings from other studies and emphasizes the need for gender-specific oral health interventions and awareness campaigns (15). The present study also identified hypertension as a significant comorbidity associated with OPMDs (OR = 2.4, *p* = 0.03). This underscores the potential role of systemic health factors in the development of OPMDs, highlighting the importance of holistic healthcare approaches in oral disease prevention and management. The association between tobacco use and OPMDs was significant. Smoking, smokeless tobacco, and combined tobacco use were all significantly associated with OPMDs. These findings corroborate and strengthen the existing body of evidence on the detrimental effects of tobacco use on oral health. Notably, **Li et al**. (20) reported a more than two-fold increase in the odds of OPMDs associated with smoking (OR 2.5, *p* < 0.001), emphasizing the significant role of smoking as a risk factor. Similarly, **Chung et al.** (21) found that individuals who were current smokers had a 4.7-fold increased risk of having an OPMD. These studies collectively highlight the consistent and robust association between smoking and OPMDs. Likewise, the present study findings are in line with the results of a comprehensive meta-analysis conducted by **Khan Z et al.** (22), which assessed the association between smokeless tobacco (SLT) products and OPMDs in 18 included studies. The pooled odds ratio (mOR) for any OPMDs with any SLT product use was 15.5, highlighting the significant risk associated with SLT. The duration of past tobacco quitting attempts demonstrated preventive association (OR = 0.7, *p* = 0.05,) with OPMDs. The present study's results are in line with previous research, which combined primary data analyses from three large cohort studies and one case-control study to investigate the relationship between the cessation of areca nut product use without tobacco and the risk of oral cancer. The data showed as the duration of cessation increased, the risk of oral cancer decreased significantly (23). This finding suggests that quitting tobacco can have a substantial impact on oral health and potentially reduce the incidence of OPMDs. India's strides in tobacco control, marked by the enactment of the Cigarettes and Other Tobacco Products Act (COTPA) in 2003, underscore its commitment to regulating tobacco production, sale, distribution, and consumption. COTPA plays a crucial role in curbing tobacco usage, especially in public places and workplaces like hospitals, where security guards operate (24). The association between tobacco usage and oral potentially malignant disorders (OPMDs) underscores COTPA's role in safeguarding public health by regulating tobacco advertising, promotion, and sponsorship. Additionally, COTPA advocates for strengthening enforcement measures to combat illicit tobacco trade and mandates pictorial health warnings on tobacco products to deter tobacco use and protect individuals from its harmful effects. Therefore, enhancing the enforcement of existing tobacco laws, such as COTPA, could prove pivotal in reducing tobacco consumption among security guards in hospital settings, thereby promoting their health and well-being.

### Strength

The study's strengths lie in its focus on security guards, an often-overlooked group, providing insights into their unique challenges. With a sizable sample of 696 participants, it offers robust statistical power for precise estimations. Comprehensive data on various risk factors, including age, gender, comorbidities, and tobacco use, enriches the exploration of relationships. The findings contribute to understanding the link between tobacco use and oral health issues, particularly in high-stress professions like security services. The study's implications extend beyond security guards, highlighting its relevance to other high-stress jobs. The findings will offer crucial insights for stakeholders, policymakers, and the general public in public health interventions aimed at reducing tobacco consumption and promoting oral health across various occupational groups.

### Limitation and recommendation

This study acknowledges a few limitations, including the cross-sectional design, which hinders causal inference and the ability to track changes in tobacco usage and oral health over time. Moreover, reliance on self-reported data may introduce recall and social desirability biases. To mitigate these biases in future research, alternative data collection methods and study designs should be considered. Objective measures, such as biochemical assays to detect tobacco metabolites, and anonymous surveys can provide more accurate and unbiased data on tobacco usage among security guards, minimizing reliance on participants’ memory and reducing the likelihood of social desirability bias, thus enhancing the validity and reliability of study findings.

Additionally, future research can incorporate comprehensive clinical assessments that include biopsies to definitively diagnose and monitor oral potentially malignant disorders (OPMDs) among security guards with different tobacco usage patterns. Furthermore, exploring the genetic and molecular factors that may predispose security guards to a higher risk of OPMDs due to tobacco use can offer insights into personalized prevention and treatment strategies.

## Conclusion

In conclusion, a high prevalence of tobacco usage (40.1%), particularly smokeless tobacco (74.5%), among security guards, emphasizes the importance of targeted interventions for tobacco cessation within this occupational group. The observed gender disparity in tobacco consumption, with a higher prevalence among male security guards (39.5%), underscores the need for gender-specific awareness campaigns and tailored tobacco cessation programs. Oral Potentially Malignant Disorders (OPMDs) were significantly more prevalent (11.4%) compared to the 1.4% observed in non-tobacco users. The association between tobacco usage and OPMDs reaffirms the well-established association between tobacco and adverse oral health outcomes. Age, gender, and comorbidities, specifically hypertension, emerged as significant factors influencing the risk of OPMDs, emphasizing the multifaceted nature of this oral health issue. The findings will offer crucial insights for stakeholders, policymakers, and the general public in public health interventions aimed at reducing tobacco consumption and promoting oral health across various occupational groups.

## Data Availability

The original contributions presented in the study are included in the article/Supplementary Material, further inquiries can be directed to the corresponding author.
